# Gut microbiota and its relation to inflammation in patients with bipolar depression: a cross-sectional study

**DOI:** 10.1186/s12991-023-00453-2

**Published:** 2023-05-19

**Authors:** Tingting Huang, Yushan Shang, Chunxiao Dai, Qixiu Zhang, Shaohua Hu, Jian Xie

**Affiliations:** 1 Department of Clinical Psychology, Affiliated Hangzhou First People’s Hospital, Zhejiang University School of Medicine, No. 261, Huansha Road, Hangzhou, 310006 China; 2 Zhejiang Provincial Clinical Research Center for Mental Disorders, Hangzhou, China; 3 Department of Psychiatry, First Affiliated Hospital, Zhejiang University School of Medicine, the Key Laboratory of Mental Disorder’s Management in Zhejiang Province, Brain Research Institute of Zhejiang University, No. 79, Qingchun Road, Hangzhou, 310003 China

**Keywords:** Bipolar disorder, Depression, Gut microbiota, Gene sequencing, Inflammation

## Abstract

**Background:**

To explore the gut microbiota characteristics in depressed patients with bipolar disorder (BD) as well as the connection between the gut microbiota and inflammatory markers.

**Methods:**

Totally 72 depressed BD patients and 16 healthy controls (HCs) were enrolled in the study. Blood and feces samples were taken from each subject. With the help of 16S-ribosomal RNA gene sequencing, the characteristics of the gut microbiota in each participant were examined. Correlation analysis was then utilized to assess the relationship between the gut microbiota and clinical parameters.

**Results:**

We found the taxonomic composition of the gut microbiota, but not its diversity, was significantly different in BD patients compared to HCs. We found the abundance of *Bacilli*, *Lactobacillales* and genus *Veillonella* were higher in BD patients than in HCs, while genus *Dorea* was more abundant in HCs. Additionally, correlation analysis showed that the bacterial genera’ abundance in BD patients was strongly correlated with the severity of depression and inflammatory markers.

**Conclusions:**

According to these results, the gut microbiota characteristics were changed in depressed BD patients, which may have been associated with the severity of depression and the inflammatory pathways.

## Background

Bipolar disorder (BD) is a severe mood disorder that results in significant deficits in social functioning and is characterized by recurring manic/hypomanic or depressive episodes [1]. Recent developments in the sciences of genetics [2], epigenetics [3], metabolomics [4], and radiomics [5] have started to reveal the complexity of BD. But there are still no clear indicators for the pathophysiology of BD, making clinical diagnosis difficult. Therefore, there is an urgent need to further explain the pathophysiology and biomarkers of BD.

Inflammatory mechanisms have been linked by prior research to the pathophysiology of BD. Serum and plasma inflammatory factors changes were observed in BD patients, such as IL-6, IL-8, TNF-α, interferon (INF)-γ and C-reactive protein (CRP) [6, 7]. Additionally, earlier research revealed that BD patients have lower levels of cytotoxic T lymphocytes (CD3 + CD8 +) [8, 9]. Increased production of inflammatory cytokines linked to brain plasticity, the hypothalamic–pituitary–adrenal (HPA) axis, and neurotransmitter metabolism may cause the start of BD [10]. It is yet unknown how these elements interact, though.

The microbiota-gut-brain (MGB) axis, a system that mediates bidirectional modulation between the brain and the gut microbiota through neuroanatomical, neuroimmune, and neuroendocrine pathways, is currently receiving increased attention in relation to mental illnesses [11, 12]. The development of mood disorders may be influenced by ongoing low-grade immune activation seen in the gut mucosal barrier [13]. Probiotic and prebiotic supplements may help to reduce inflammatory reactions and encourage the restoration of intestinal barrier function [13]. For instance, recent research has shown that giving probiotics to mice for five weeks reduces depressive-like behavior and TNF and IL-6 levels [14]. Maternal-separated rats with Bifidobacteria-treated exhibited a lower degree of anxiety in the swimming and climbing tests [15]. Notably, germ-free mice showed increased depressive-like behaviors after receiving fecal transplantation from depressed patients [16]. The brain-gut-microbiota axis has so far provided preliminary evidence from ongoing studies that the gut microbiota plays a significant role in BD [17]. Given that the gut microbiota has been linked to immune function, gut dysbiosis may be a significant component in BD patients' immunological failure [18].

Therefore, this preliminary study aimed to investigate the gut microbiota characteristics in depressed BD patients and healthy controls (HCs), and its relation to inflammation and clinical parameters.

## Methods

### Participants

In the study, drug-naive BD patients with depressive episodes from the Psychiatry Department of the First Affiliated Hospital of Zhejiang University were enrolled in the study. As HCs, healthy volunteers from the neighborhood were enlisted. All participants have signed the written informed consent for this study. All depressed BD patients' clinical data was gathered by a psychiatrist. The diagnosis was obtained using the Mini International Neuropsychiatric Examination (M.I.N.I.) [19], a structured psychiatric interview in accordance with DSM-IV-TR criteria. BD individuals with any additional psychiatric or severe physical conditions were disqualified. Additionally, all individuals were prohibited from using probiotics, prebiotics, or antibiotics within 4 weeks. All female contestants who were pregnant or nursing were disqualified.

### Clinical data collection

Demographic and clinical data of all participants, including sex, age, body mass index (BMI), and onset age, duration of illness, disease type, and family history, were collected. The Montgomery-Asberg Depression Rating Scale (MADRS) [20] was used to evaluate patients' depressive symptoms clinically. The Young Mania Rating Scale (YMRS) was used to define the severity of mania [21].

### Plasma cytokines and T lymphocytes level determination

Venous blood samples (1 mL) were collected from all patients between 8:30 a.m. and 10:30 a.m. after visiting the hospital. The lymphocyte subsets were identified using 50uL blood samples, and the remaining blood was separated within 15 min, with the plasma being stored at − 80 °C. The plasma levels of cytokines (IL-2, IL-4, IL-6, IL-10, IL-17A, and TNF-α) were determined by a BD cytometric bead array (CBA) kit, the human Th1/Th2/Th17 kit (BD Biosciences, CA, USA), according to the manufacturer's instructions. Briefly, the CBA assay contained six beads coated with capture antibodies, and each bead had different fluorescence intensities. The cytokine capture beads were mixed with test samples, antibodies on the cytokine capture beads combined with the corresponding antigen or protein in the samples, and then incubated with the phycoerythrin-conjugated detection antibodies to form sandwich complexes. Sample data were obtained and analyzed using FACS Calibur™ flow cytometer (BD Biosciences, CA, USA) and BD CBA Software (BD Biosciences, CA, USA). Each plasma cytokine was given its own standard curve. The minimum limit of detection for all the cytokines was 0.1 pg/ml. The CRP level was measured by enzyme-linked immunosorbent assay (ELISA) using the Human CRP ELISA kit (R&D Systems, Minnesota, USA) following the manufacturer’s instructions. The following antibodies were used for flow cytometry analysis of lymphocyte subsets (CD3, CD4, CD8, and natural killer [NK] cells): Pcy5-conjugated anti-CD3, FITC-conjugated anti-CD4, P-phycoerythrin-conjugated anti-CD8, and PE-conjugated anti-CD16/CD56 (BD Biosciences, CA, USA) (Beckman Coulter, CA, USA). Blood samples (50 µL) were added to a tube containing 10 µL of each antibody, mixed in the dark and incubated at room temperature for 20 min. Then red blood cells were lysed and the cells were fixed. Data on a minimum of 20,000 lymphocytes were counted and analyzed using the BD FACS Calibur™ flow cytometer (BD Biosciences, CA, USA) and BD CellQuest Pro software (BD Biosciences, CA, USA).

### Fecal sample collection and gene sequencing

Fecal samples from all subjects were collected and divided into 0.2 g each, then stored at -80 °C within half an hour after collection. DNA was extracted using the PSP® Spin Stool DNA Plus kit following the manufacturer’s instructions. The degree of DNA degradation and potential contamination was estimated using agarose gel electrophoresis. The extracted DNA was diluted to 10 ng/μL for microbial analysis after being quantified with a Qubit® Fluorometer.

The V3–V4 variable region in the 16S rRNA gene was selected, and the primers were 341F 5′-barcode-CCTACGGGNGGCWGCAG-3′ and 785R 5′-GACTACHVGGGTATCTAATCC-3′ for PCR assay. 2 µl PCR products were taken for 2% agarose gel electrophoresis detection after the first round PCR amplification, then purified using AMPure XP Beads. In the second round PCR amplification, the target fragments were cut and recovered, and the library was purified and then checked for library quality. The high-throughput sequencing was performed using an Illumina MiSeq platform according to the manufacturer’s instructions.

Clusters randomly selected sequences from all samples into operational taxonomic units (OTU) using the "Usearch -cluster_otus" function with default parameters. OTU profiles were constructed by aligning randomly picked sequences with a representative OTU sequence as a reference using the "Usearch -usearch_global" function with a 97% cutoff. The relative abundance of OTU was used to calculate the alpha diversity and beta diversity, and to analyze the microbiota with statistically significant differences in relative richness between the two groups.

### Statistics analysis

Clinical data analysis was conducted using the SPSS 20.0 statistical software (IBM, IL, USA). Measurement data was expressed as "mean ± standard deviation ($\bar{\text{x}}$ ± S)". The alpha diversity was calculated using the Wilcoxon rank sum test. The difference in gut microbiota between BD patients and HCs was compared using linear discriminant analysis (LDA), with an LDA score (log10) = 2 serving as the crucial value. The correlations between gut microbiota and clinical indicators in BD patients were analyzed using Pearson/Spearman correlation. *P* < 0.05 was used to indicate a statistical difference.

## Results

### Clinical characteristics

Finally, 72 depressed BD patients and 16 HCs were recruited in the study. Compared to HCs, there was no discernible difference in the sex and BMI values in BD patients. The two groups did differ in terms of age, with HCs being more likely to be older (*P* < 0.05). Table 1 displays certain attributes.

**Table 1:** Demographic and Clinical Details of Recruited Subjects

Demographic and Clinical Indexes	BD		HCs		*P*
	*N* = 72	**%**	*N* = 16	**%**	
Sex					
Female	33	47.14	9	56.25	0.59
Male	37	52.86	7	43.75	
Age (year, mean ± SD)	24.16 ± 9.26		42.75 ± 11.22		< 0.001
BMI (kg/m^2^, mean ± SD)	21.39 ± 9.32		21.89 ± 12.29		0.60
MARDS Scores (mean ± SD)	25.93 ± 10.18		0		< 0.001
YMRS Scores (mean ± SD)	0.45 ± 0.80		0.44 ± 0.73		0.81
Onset age (year, mean ± SD)	19.29 ± 7.58		–		–
Duration of illness (year, mean ± SD)	4.92 ± 5.63		–		–
Bipolar diagnosis					
I	13	18.06			
II	45	62.50			
Other	14	19.44			
Family history					
Yes	16	22.22			
No	44	61.11			
Unknown	12	16.67			

*BD* Bipolar disorder; *HCs* Healthy controls; *BMI* Body Mass Index; *MARDS* Montgomery-Asberg Depression Rating Scale; *YMRS* Young Mania Rating Scale

### Inflammatory profile changes in BD patients

Regarding to inflammatory profiles, significant differences were found in serum IL-6, TNF-α and CRP levels between BD patients and HCs. While no significant difference was found in IL-2, IL-4, IL-10, IL-17A and CD3, CD4, CD8, NK levels. Details are shown in Table 2.

**Table 2:** Serum Inflammatory Factors in BD patients and HCs

Inflammatory factors	BD	HCs	*P*
	*N* = 72	*N* = 16	
IL-2 (pg/ml, mean ± SD)	0.41 ± 0.25	0.74 ± 0.52	0.29
IL-4 (pg/ml, mean ± SD)	0.15 ± 0.17	0.18 ± 0.82	0.69
IL6 (pg/ml, mean ± SD)	17.72 ± 42.97	0.43 ± 0.38	0. 007
IL-10 pg/ml, (mean ± SD)	0.94 ± 1.47	0.95 ± 1.40	0.99
IL-17A (pg/ml, mean ± SD)	3.13 ± 9.39	0.99 ± 2.86	0.31
TNFα (pg/ml, mean ± SD)	1.89 ± 4.34	0.47 ± 0.44	0.045
CRP (mg/l, mean ± SD)	1.77 ± 2.87	0.32 ± 0.38	0.025
CD3 (%, mean ± SD)	65.86 ± 12.66	62.60 ± 12.73	0.34
CD4 (%, mean ± SD)	33.50 ± 9.33	33.05 ± 9.66	0.82
CD8 (%, mean ± SD)	27.82 ± 9.69	26.56 ± 9.17	0.62
NK (%, mean ± SD)	14.59 ± 6.82	17.91 ± 9.50	0.11

*BD* Bipolar disorder, *HCs* Healthy controls, *CRP* C-reactive protein, *INF* interferon, *NK* natural killer

### Changes of gut microbiota diversity in BD patients

**Fig. 1:**
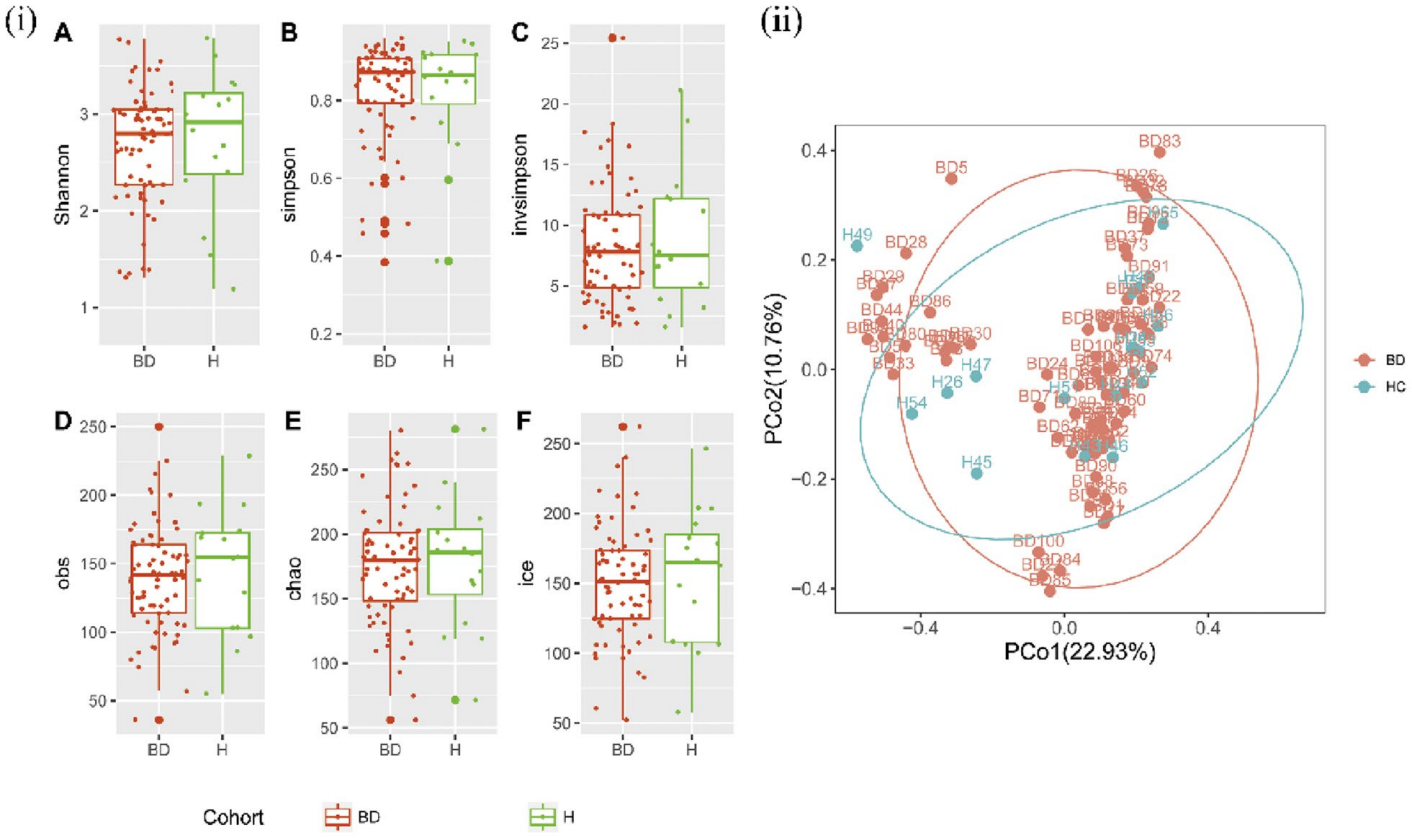
Gut microbiota diversity in BD patients and HCs. (i) There was no significant difference in BD patients and HCs according to the α-diversity index (Shannon, Simpson, invsimpson, obs, chao1 and ice index). (ii) PCoA at OTU level showed that no significant difference was found in two groups

In the study, the changes in gut microbiota diversity were assessed by the α-diversity and the β-diversity indices. Several α-diversity indexes, including Shannon, Simpson, inverse Simpson [invSimpson], Obs, Chao and Incidence-based Coverage Estimators [ICE]) revealed no significant difference in gut microbiota α-diversity between BD patients and HCs (Fig. 1i). The gut microbiota β-diversity was used to analyze the similarities and differences of gut microbiota in depressed BD patients and HCs. Principal coordinate analysis (PCoA) was performed to compare the phylogenetic similarity distance of microbial communities. There was no significant difference between depressed BD patients and HCs (Fig. 1ii).

### Changes of gut microbiota taxonomic composition in BD patients

**Fig. 2:**
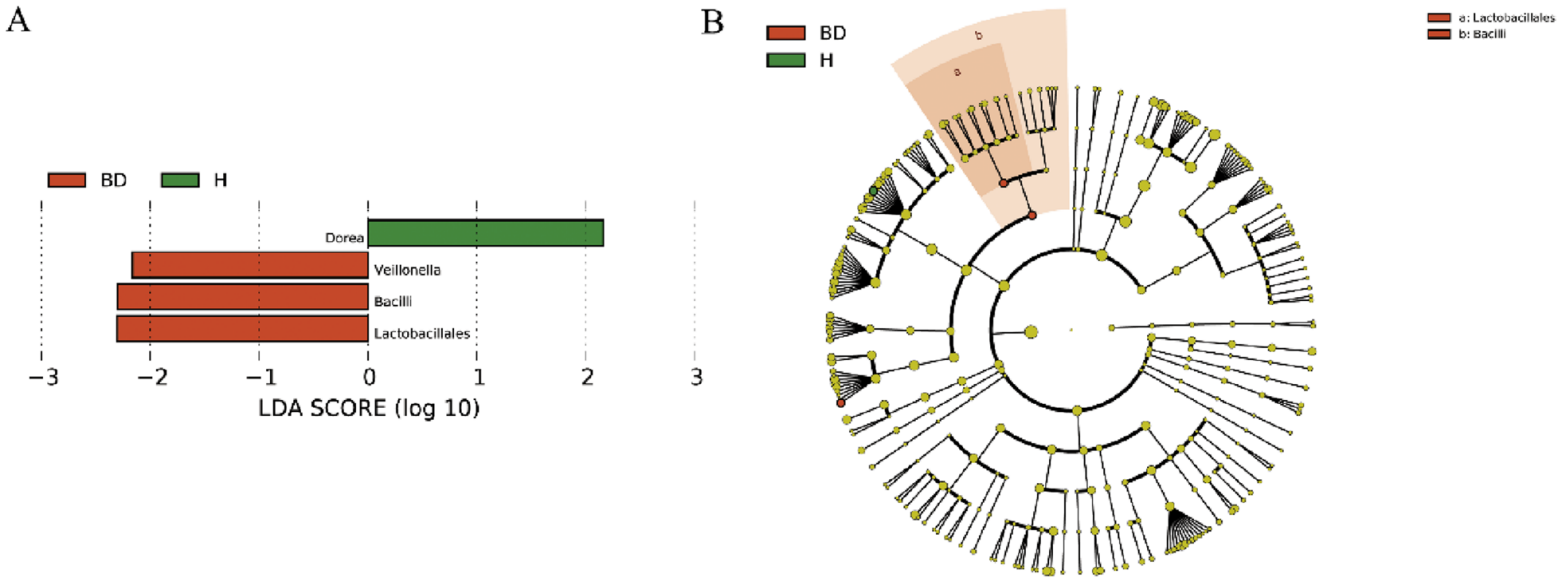
Gut microbiota taxonomic composition changes in BD patients and HCs. Compared to HCs, the abundance of Bacilli, Lactobacillales and Veillonella was increased, while genus Dorea was decreased (*P* < 0.05, LDA score > 2)

According to the taxonomic information of bacteria, the bacterial species were carried out at the five levels of phylum, class, order, family and genus. Respectively, 97.6% and 91.03% of all reads could be assigned to family and genus levels. Lefse analysis showed that compared with HCs, the abundance of *Bacilli*, *Lactobacillales* and *Veillonella* was increased, while the genus *Dorea* was more abundant in HCs (*P* < 0.05, LDA score > 2) (Fig. 2).

### Associations of gut microbiota with clinical parameters

**Fig. 3:**
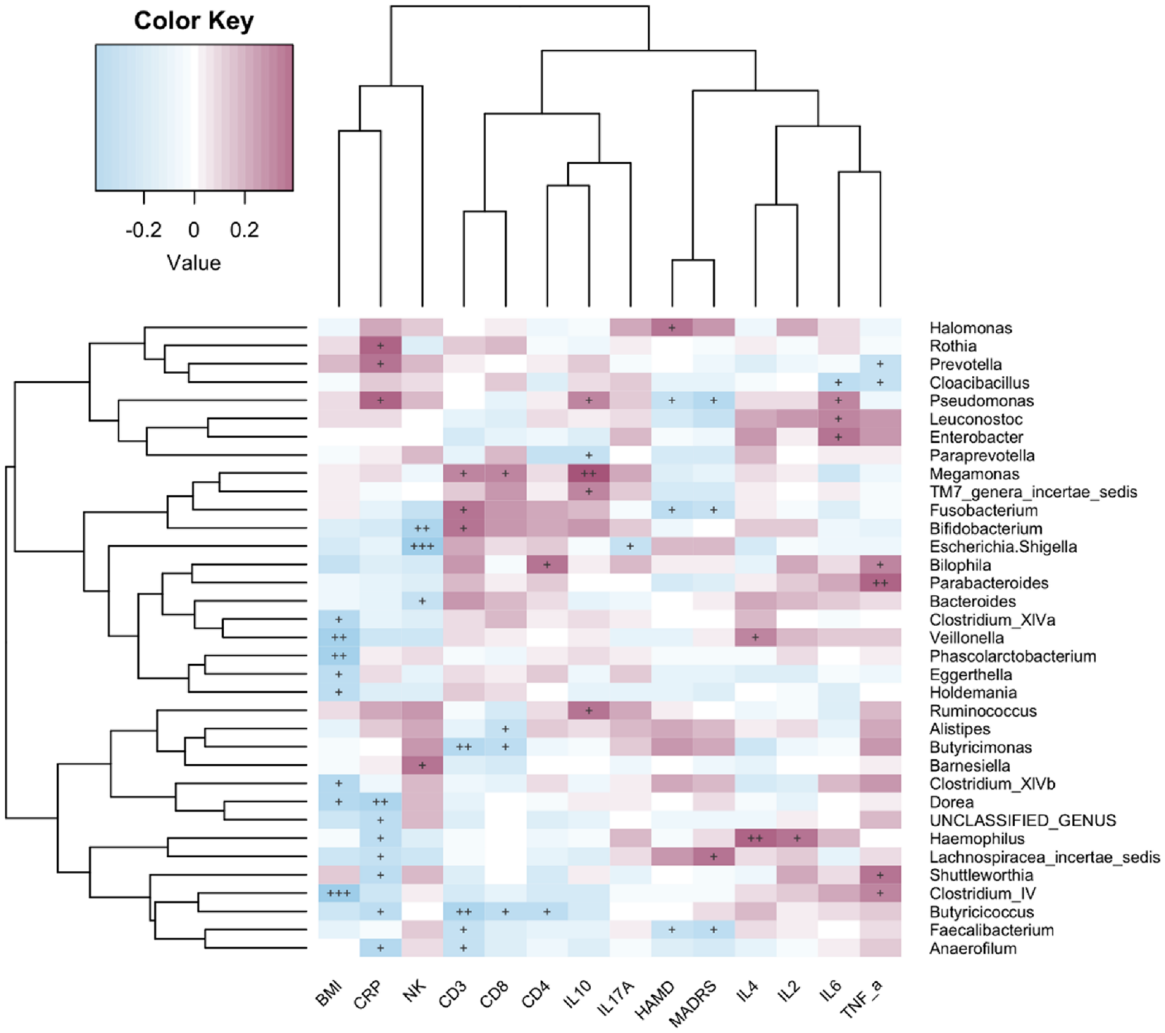
Associations between gut microbiota and clinical parameters. Heat map revealed that gut microbiota was closely associated with severity of depression and inflammatory markers in depressed BD patients (*P* < 0.05). Red and blue edges denoted Spearman’s rank correlation coefficient > 0.2 and <  − 0.2, respectively

**Fig. 4:**
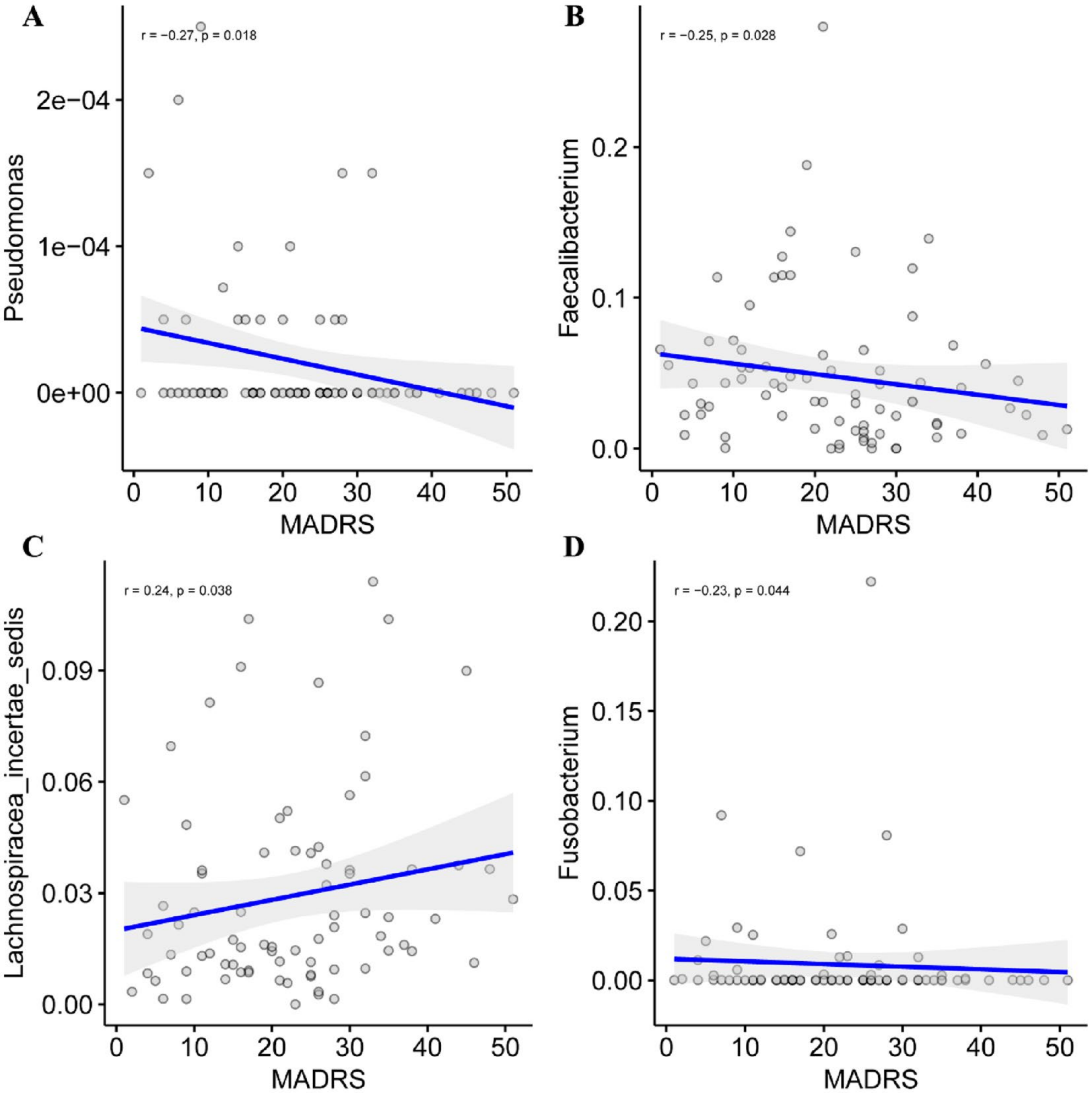
Association between gut microbiota and MADRS scores in BD patients. Pairwise correlation of MADRS with the abundance of Pseudomonas, Faecalibacterium, Lachnospiracea_incertae_sedis and Fusobacterium (*P* < 0.05)

**Fig. 5:**
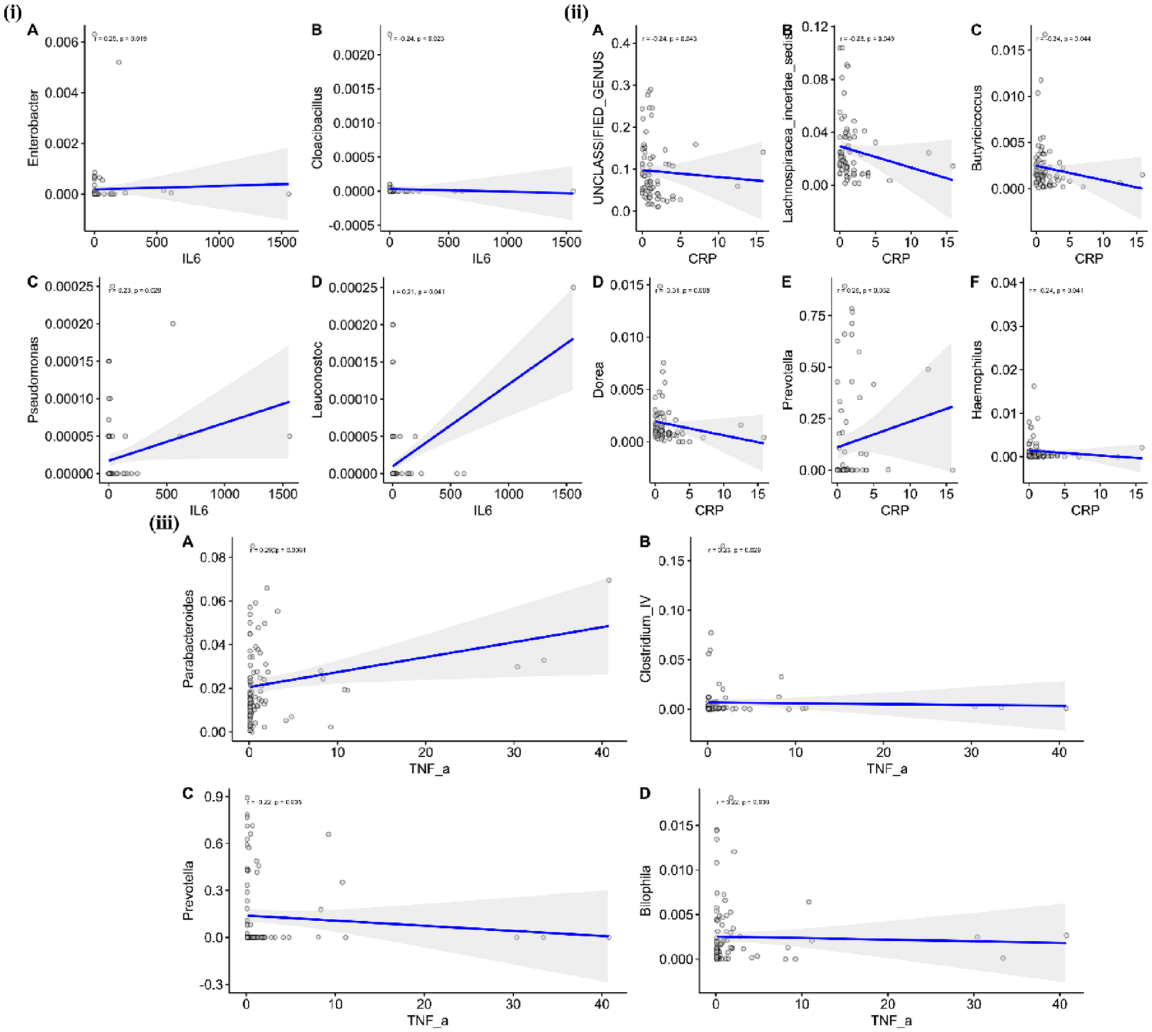
Association between gut microbiota and inflammatory markers in BD patients. (i) Serum levels of IL-6 were positively correlated with Enterobacter, Pseudomonas and Leuconostoc abundance, but negatively associated with Cloacibacillus abundance (*P* < 0.05). (ii) Serum CRP levels were positively correlated with Prevotella abundance, but negatively associated with Butyricicoccus, Lachnospiraceae incertae sedis and Dorea abundance (*P* < 0.05). (iii) TNF-α levels correlated positively with Parabacteroides, Clostridium IV and Bilophila abundance but negatively with Prevotella abundance (*P* < 0.05)

We assessed the relationship between gut microbiota and clinical parameters. The severity of depression and inflammatory markers were closely associated with the abundance rates of bacterial genera in depressed BD patients (Fig. 3). MADRS scores were positively correlated with *Lachnospiracea_incertae_sedis* abundance, but negatively correlated with *Faecalibacterium*, *Pseudomonas* and *Fusobacterium* abundance (*P* < 0.05; Fig. 4). Serum levels of IL-6 were positively correlated with *Enterobacter*, *Pseudomonas* and *Leuconostoc* abundance, but negatively associated with *Cloacibacillus* abundance (*P* < 0.05). TNF-α levels correlated positively with *Parabacteroides*, *Clostridium* IV and *Bilophila* abundance but negatively with *Prevotella* abundance (*P* < 0.05). Furthermore, Serum CRP levels were positively correlated with *Prevotella* abundance, but negatively associated with *Butyricicoccus*, *Lachnospiraceae incertae sedis* and *Dorea* abundance (*P* < 0.05; Fig. 5).

## Discussion

Our study used high-throughput sequencing technology to explore the gut microbiota characteristics in depressed BD patients and the relationship between gut microbiota and inflammatory markers. The findings indicated that the taxonomic composition of the gut microbiota, but not its diversity, was significantly different in BD patients compared to HCs. Additionally, the abundance of several genera was correlated with inflammatory factors and depressive severity.

We found that there was a higher abundance of *Dorea* in healthy individuals, while a greater abundance of *Veillonella* was found in BD patients. This finding appears to be in line with previous studies. *Blautia*, *Fecalibacterium*, and *Dorea* were found to be related to cognition function and could alleviate the inflammatory reaction in cirrhosis patients [22]. *Veillonella* levels have been found to be higher in patients with irritable bowel syndrome [23], and the lipopolysaccharide (LPS) produced by *Veillonella* could stimulate the release of TNF-α, IL-1, IL-6 and IL-10 within Toll-like receptor (TLR) pathways [24]. As a result, *Veillonella* might participate in the brain-gut axis through inflammatory mechanisms.

Our study found that the abundance of *Faecalibacterium*, *Pseudomonas* and *Fusobacterium* was negatively correlated with the severity of depression. Previous studies have reported consistent conclusions that the negative association between *Faecalibacterium* abundance and depression severity in depressive and BD patients [25, 26]. *Faecalibacterium* was found to produce a protein which was involved in anti-inflammatory activities [27]. Notably, as a member of the Firmicutes phylum, *Faecalibacterium* was found to be important for the biosynthesis of the microbial product butyrate, which may be beneficial for immune accommodation, gut barrier regulation, gut metabolism, and energy modulation in the gut [28, 29]. Furthermore, butyrate in the central nervous system could influence the function of the hippocampus and facilitate upregulation of BDNF, which may have an antidepressant-like effect in animal models [30, 31]. *Fusobacterium*, which belongs to the *Fusobacteria* phylum, was also identified as one of the potential butyrate producers, which could modulate the gut microbiota and even modify anti-inflammatory, tumor-fighting, and metabolic pathways [32, 33]. Hence, low abundance of *Faecalibacterium*, *Pseudomonas* and *Fusobacterium* in depressed BD patients may have implications for the neurobiology of BD.

BD is closely associated with immune dysfunction, and a higher level of pro-inflammatory factors has been found in BD patients [34]. One study examined gut microbiota and its relationship to inflammation in BD patients and discovered that changes in gut microbiota in BD patients could be a factor contributing to immune alterations [35]. In the current study, serum IL-6 levels were found to be positively correlated with *Enterobacter*, *Pseudomonas* and *Leuconostoc* abundance, but negatively correlated with *Cloacibacillus* abundance in BD patients. *Enterobacter* belongs to the Enterobacteriaceae family and is enriched in aged individuals [36]. Compared with healthy controls, it has been previously reported that the abundance of Enterobacteriaceae and *Alistipes* was increased, and *Faecalibacterium* abundance was decreased in patients with major depressive disorder [26]. Our findings are the first to report the association between *Cloacibacillus,* an amino-acid-fermenting bacterium [37], and IL-6 in BD patients. In addition, TNF-α was found to be positively correlated with the abundance of *Parabacteroides*, *Bilophila*, and Clostridium IV, but negatively correlated with the abundance of *Prevotella*. When compared to non-stressed control mice, the abundance of Parabacteroides was found to be increased in stressor-exposed mice, as were the circulating levels of TNF-α and TNF-γ [38]. However, the relationship between the bacteria and these cytokine levels was not investigated in this study [38]. In contrast, another study showed that oral administration of *Parabacteroides distasonis* could reduce the production of TNF-α [39]. In healthy individuals, *Bilophila* was negatively correlated with LPS-induced production of TNF-α [40]. This is inconsistent with our findings in BD patients. Acute stress could reduce *Prevotella* abundance and result in an inflammatory response [41]. In our study, serum CRP concentration was positively correlated with *Prevotella* abundance, but negatively correlated with *Butyricicoccus*, *Lachnospiraceae incertae sedis* and *Dorea* abundance in BD patients. Similar results have been reported in obese adolescents where the abundance of *Prevotella* was positively associated with serum levels of triglycerides (TG) and high-sensitive CRP (hs-CRP) [42]. As a butyrate-producing bacteria, *Butyricicoccus* was positively correlated with anti-inflammatory cytokines such as IL-4, IL-10, and IL-11 [43]. The negative association between CRP and *Butyricicoccus* abundance may reflect an imbalance between pro- and anti- inflammatory reactions.

Several limitations of the study need to be pointed out. The influence of diet and region was not evaluated in this study. Although no significant difference was found in terms of gender and BMI between BD patients and controls, the diet and age of participants were not well controlled. Finally, the sample size in HCs was relatively small.

## Conclusions

This study suggests that there are significant alterations in the taxonomic compositions of the gut microbiota in depressed BD patients, which may be related to inflammatory pathways and depression severity. The study provides preliminary evidence that the gut microbiota and inflammatory processes may have an impact on the neurobiology of BD.

## Data Availability

The datasets used and analyzed during the current study are available from the corresponding author on reasonable request.

## Abbreviations

BD: Bipolar disorder
CRP: C-reactive protein
HPA: Hypothalamic–pituitary–adrenal
MGB: Microbiota-gut-brain
HCs: Healthy controls
MINI: Mini International Neuropsychiatric Interview
MADRS: Montgomery-Asberg Depression Rating Scale
BMI: Body mass index
YMRS: Young Manic Rating Scale
CBA: Cytometric bead array
ELISA: Enzyme-linked immunosorbent assay
NK: Natural killer
invSimpson: Inverse Simpson
ICE: Incidence-based Coverage Estimators
PCoA: Principal coordinate analysis
LPS: Lipopolysaccharide
TLR: Toll-like receptor
LDA: Linear discriminant analysis
Lefse: LDA effect size
hs-CRP: High-sensitive CRP
